# Hemostatic capability of ultrafiltrated fresh frozen plasma compared to cryoprecipitate

**DOI:** 10.1038/s41598-023-48759-1

**Published:** 2023-12-07

**Authors:** Junko Ichikawa, Toshiaki Iba, Ryouta Okazaki, Tomoki Fukuda, Mitsuharu Kodaka, Makiko Komori, Jerrold H. Levy

**Affiliations:** 1 Department of Anesthesiology, Tokyo Women’s Medical University, Adachi Medical Center, 4-33-1 Kouhoku, Adachi-ku, Tokyo, 123-8858 Japan; 2 Department of Emergency and Disaster Medicine, Juntendo University Graduate School of Medicine, Tokyo, Japan; 3 Department of Anesthesiology, Critical Care, and Surgery, Duke University School of Medicine, Durham, NC USA

**Keywords:** Medical research, Nephrology

## Abstract

This in vitro study evaluated the potential hemostatic effect of fresh frozen plasma (FFP) ultrafiltration on clotting factors, coagulation parameters, and plasma properties. ABO-specific units of FFP (n = 40) were prepared for the concentrated FFP and cryoprecipitate. Plasma water was removed from FFP by ultrafiltration using a dialyzer with a pump running at a 300 mL/min. The aliquot of each concentrated FFP after 50, 100, 200, and 250 mL of fluid removal were measured the standard coagulation assay, clotting activity, and plasma properties to compare those parameters of cryoprecipitate. Concentrated FFP contained 36.5% of fibrinogen in FFP with a mean concentration of 7.2 g/L, lower than the cryoprecipitate level. The levels of factor VIII (FVIII), von Willebrand factor (VWF):antigen (Ag), and VWF:ristocetin cofactor (RCo) were also lower in concentrated FFP, whereas the levels of factor V, factor IX, factor XIII, antithrombin and albumin was higher in concentrated FFP. Maximum clot firmness (MCF) in thromboelastometry was approximately one-half of that in cryoprecipitate. Although the levels of VWF:Ag, VWF:RCo, and FVIII differed depending on the ABO blood types, fibrinogen levels, and MCF were not significantly different among the ABO blood groups in FFP and concentrated FFP.

## Introduction

Unexpected bleeding and subsequent hemostatic impairment are common following cardiac surgery. Hemostasis critically depends on fibrinogen as a substrate for clot formation and as the ligand for platelet aggregation^1,2^. In clinical settings, there is divergent practice regarding the preferred product for fibrinogen replacement. For example, the European guidelines^3^ suggest fibrinogen concentrates for the primary treatment of acquired hypofibrinogenemia, and in cases where fibrinogen concentrates are not available, cryoprecipitate is the choice. Cryoprecipitate contains a concentrated source of fibrinogen but also significant amounts of factors VIII (FVIII), XIII (FXIII), von Willebrand factor (VWF), and fibronectin^4^. However, cryoprecipitate from blood centers is only supplied in a limited number of countries such as the USA and UK. In other countries, including Japan, locally prepared “in-house” cryoprecipitate is the choice, and it is not consistently available. Another issue of in-house cryoprecipitate preparation is the quality control between the institutions^5^. If in-house cryoprecipitate is not available, fresh frozen plasma (FFP) is the only choice. FFP contains all plasma coagulation factors and anticoagulants; however, a large amount of FFP is required to increase fibrinogen levels^6^, which may lead to volume overload and hemodilution^7^. While the supplementation of fibrinogen is crucial in various situations, there is limited recognition of the differences in content, volume, and fibrinogen content between fibrinogen concentrates, cryoprecipitate, and FFP. We believe it is essential to comprehend these distinctions for appropriate usage.

Ultrafiltration (UF) is used to manage fluid overload, preserve fibrinogen, and other coagulation factors during cardiac surgery^8^. Modified ultrafiltration (MUF), performed after separation from cardiopulmonary bypass (CPB) and before decannulation, has been adopted as the standard of care in pediatric patients^9^. In large series of unselected adult patients, MUF use was shown to be associated with a lower prevalence of early morbidity and lower blood transfusion requirements^10^. MUF’s potential benefits include tissue edema reduction, hemoconcentration, and removal of inflammatory mediators^8–10^. Therefore, we hypothesized that concentrated FFP following UF administered in the CPB circuit also could attenuate the volume effect of FFP and increase the functional fibrinogen in cardiovascular surgery. As a result, we examined the hemostatic potential of FFP following UF and the differences between concentrated FFP and cryoprecipitate.

## Results

Table 1 demonstrates the pre- and post-UF trends of coagulation factors, coagulation parameters, plasma proteins, and liquid properties after removing predefined volume of fluid. Although the levels of all measured coagulation factors and anticoagulants, protein, and density increased significantly by the plasma concentration, the increases were not parallel with the concentrated volume. The mean fibrinogen, FV, FVIII, FIX, FXIII, VWF:RCo, and albumin level increased by 170%, 178%, 190%, 182%, 114%, 148%, and 144%, respectively, in the final concentrate after UF compared with FFP. The mean MCF in EXTEM and NATEM significantly increased from 21.1 to 36.5 mm (76%) and 22.1 to 34.4 mm (59%), respectively, in aliquot from FFP and post-UF after removing 250 mL of fluid. CT in NATEM and CFT were significantly shortened in concentrated FFP than in FFP, whereas CT in EXTEM was significantly elevated after removing 200 mL of fluid. Alpha angles in NATEM were approximately 66% higher in concentrated FFP after removing 250 mL of fluid than in FFP (*p* < 0.0001). The process lasted a mean of 32 min, during which the FFP was recirculated through the circuit approximately 6.9 times. In the process of UF, the filtration time got longer as removal volume increased.

**Table 1:** Trends of activity of clotting factors and coagulation petameters, plasma protein and plasma properties from FFP to each hemofiltration.

Coagulation factors	FFP	50 mL removed	100 mL removed	200 mL removed	250 mL removed
Factor VIII (%)	108.7 ± 32.6	158.6 ± 60.0	194.1 ± 76.9*	264.8 ± 78.8*	308.7 ± 79.1*
Factor V (%)	92.0 ± 16.9	128.8 ± 25.8*	149.1 ± 34.6*	209.8 ± 49.6*	256.3 ± 61.6*
Factor IX (%)	108.6 ± 14.5	154.2 ± 25.2*	177.7 ± 28.6*	248.0 ± 42.7*	305.1 ± 54.8*
Factor XIII (%)	94.7 ± 18.5	130.6 ± 25.7*	143.7 ± 28.4*	192.0 ± 50.9*	213.7 ± 53.7*
VWF:RCo (IU/dL)	93.6 ± 26.2	124.5 ± 37.8	146.4 ± 41.8*	187.1 ± 50.9*	229.6 ± 71.8*
VWF:Ag (IU/unit)	113.8 ± 37.8	157.4 ± 62.8	188.1 ± 78.8*	251.5 ± 82.7*	275.7 ± 90.4*
Osmotic pressure (mOsm/kg/H_2_O)	288.2 ± 3.1	273.6 ± 6.5*	270.6 ± 3.4*	271.8 ± 3.2*	271.5 ± 9.3*
PT (s)	12.2 ± 0.6	11.4 ± 0.4*	11.3 ± 0.5*	11.0 ± 0.4*	11.1 ± 0.5*
APTT (s)	28.3 ± 2.2	27.6 ± 2.3	27.4 ± 2.4	27.5 ± 2.4	27.9 ± 2.4
Fibrinogen (mg/dL)	273.0 ± 69.7	388.1 ± 68.7*	454.5 ± 84.9*	631.1 ± 118.0*	716.9 ± 143.8*
Antithrombin (%)	96.4 ± 12.4	128.3 ± 15.8*	140.5 (14.8) *	> 150*	> 150*
Total protein (g/dL)	6.2 ± 0.3	8.3 ± 0.9*	9.5 ± 1.1*	13.1 ± 2.1*	15.9 ± 2.1*
Albumin (g/dL)	3.6 ± 0.3	5.0 ± 0.7*	5.8 ± 0.9*	7.7 ± 0.8*	8.8 ± 1.0*
The time during each filtration (min)	NA	279.7 ± 49.5	355.0 ± 85.4	813.6 ± 234.1	380 (273.8)
Plasma density	1.023 ± 0.001	1.029 ± 0.003*	1.033 ± 0.004*	1.041 ± 0.004*	1.047 ± 0.005*
EXTEM					
CT	59.3 ± 4.7	65.5 ± 13.0	70.1 ± 10.0	79.3 ± 22.0*	86.4 ± 14.6*
CFT	NA	195.6 ± 207.7	117.1 ± 122.9	72.1 ± 34.1	69.1 ± 16.3
α	77.9 ± 2.5	79.4 ± 2.3	79.5 ± 1.7	77.1 ± 7.9	75.8 ± 4.3
MCF	21.1 ± 4.8	27.2 ± 4.7*	27.6 ± 4.9*	35.5 ± 5.7*	36.5 ± 8.2*
NATEM					
CT	824.4 ± 274.1	768.1 ± 198.7	703.3 ± 178.6	673.6 ± 193.3	650.7 ± 119.1*
CFT	900.4 ± 457.3	248.0 ± 140.9*	267.3 ± 167.0*	157.5 ± 94.0*	154.2 ± 63.5*
α	40.1 ± 13.2	55.7 ± 10.8*	54.1 ± 13.4*	62.1 ± 12.6	63.2 ± 8.4*
MCF	22.1 ± 5.4	26.9 ± 3.6*	27.8 ± 4.7*	33.8 ± 4.5*	34.4 ± 5.8*

Values are expressed as mean ± SD or median (range). Differences compared to baseline (*) are shown if results from two-way analysis of variance for repeated measures are significant (P < 0.05).

*FFP* fresh frozen plasma, *VWF:RCo* von Willebrand factor ristocetin cofactor activity, *VWF:Ag* von Willebrand factor antigen, *PT-INR* prothrombin time-international normalized ratio, *PT* prothrombin time, *APTT* activated partial thromboplastin time, *EXTEM* extrinsic test, *CT* clotting time, *CFT* clot formation time, *MCF* maximum clot firmness, *NATEM* non-activated thromboelastometry, *NA* not assessed.

Table 2 demonstrates the levels of coagulation parameters, plasma proteins, density, osmotic pressure, and volume between cryoprecipitates and concentrated plasma products. UF resulted in concentrating mean plasma volume of 481.6 mL by an order of 6.4 to 19.3 times. After UF, mean plasma volume was 57.3 mL, which was not significantly different from cryoprecipitate volume. The fibrinogen concentration in concentrated FFP ranged from 4.2 to more than 9.0 g/L (n = 4), with a mean concentration of 7.2 g/L, which was significantly lower than that in cryoprecipitate units. Cryoprecipitates contained more VWF and FVIII than the concentrated FFP, whereas FV, FIX, FXIII, and antithrombin values were significantly higher in concentrated FFP than those in cryoprecipitate units. In addition, total protein, albumin levels, and density were significantly higher in the concentrate after UF than in cryoprecipitate, while osmotic pressure was significantly lower in concentrated FFP than in cryoprecipitate units.

**Table 2:** Activity of clotting factors and coagulation petameters, plasma protein and plasma properties in cryoprecipitates and ultrafiltered FFP units.

Coagulation factors	Cryoprecipitate	Ultrafiltration	p values
VWF-RCo (IU/dL)	615.2 ± 176.6	229.6 ± 71.8	< 0.01
VWF:Ag (IU/unit)	865.6 ± 284.3	275.7 ± 90.4	< 0.01
Factor V (%)	65.4 ± 11.6	256.3 ± 61.6	< 0.01
Factor IX (%)	129.3 ± 26.3	305.1 ± 54.8	< 0.01
Factor VIII (%)	486.9 ± 182.7	308.7 ± 79.1	< 0.01
Factor XIII (%)	176.2 ± 49.6	213.7 ± 53.7	0.028
Fibrinogen (mg/dL)	> 900	716.9 ± 143.8	< 0.01
PT (s)	13.7 ± 0.66	11.1 ± 0.5	< 0.01
APTT (s)	26.9 ± 2.3	27.9 ± 2.4	0.174
Antithrombin (%)	89.5 ± 10.5	> 150	< 0.01
Total protein (g/dL)	7.3 ± 0.6	15.9 ± 2.1	< 0.01
Albumin (g/dL)	3.6 ± 0.3	8.8 ± 1.0	< 0.01
Osmotic pressure (mOsm/kg/H_2_O)	304.0 ± 19.1	271.5 ± 9.3	< 0.01
Volume (mL)	52.1 ± 3.4	57.3 ± 11.6	0.07
Plasma density	1.03 ± 0.002	1.047 ± 0.005	< 0.01
EXTEM			
CT (s)	54.7 ± 6.0	86.4 ± 14.6	< 0.01
CFT (s)	22.9 ± 3.5	69.1 ± 16.3	< 0.01
Alpha angle	85.7 ± 0.6	75.8 ± 4.3	< 0.01
MCF (mm)	65.0 ± 10.0	36.5 ± 8.2	< 0.01
NATEM			
CT (s)	718.5 ± 104.9	650.7 ± 119.1	0.067
CFT (s)	193.4 ± 88.9	154.2 ± 63.5	0.123
Alpha angle	64.2 ± 8.4	63.2 ± 8.4	0.729
MCF (mm)	66.7 ± 10.4	34.4 ± 5.8	< 0.001

Values are expressed as mean ± SD.

*VWF:RCo* von Willebrand factor ristocetin cofactor activity, *VWF:Ag* von Willebrand factor antigen, *PT-INR* prothrombin time-international normalised ratio, *PT* prothrombin time, *APTT* activated partial thromboplastin time, *EXTEM* extrinsic test, *CT* clotting time, *CFT* clot formation time, *MCF* maximum clot firmness, *NATEM* non-activated thromboelastometry.

MCF in EXTEM and NATEM were approximately two-fold higher in cryoprecipitate units than in concentrated FFP. CT and CFT in EXTEM were significantly shortened in cryoprecipitate units than in concentrated FFP (*p* < 0.001), but the difference was not observed in CT and CFT in NATEM. Alpha angles in EXTEM were significantly higher in cryoprecipitate samples than in concentrated samples (*p* < 0.01). Neither NATEM nor EXTEM showed any sign of fibrinolysis based on ML > 15%^11^. The post hoc power analysis showed that a sample size of 20 per unit was adequate with 100% power. The MCF's effect size (f) in EXTEM and NATEM were 4.6 and 3.6, respectively, with an α-level of 0.05.

Table 3 demonstrates a subset analysis of the levels of fibrinogen, FVIII, VWF, and ROTEM-MCF in FFP, cryoprecipitate, and concentrated FFP from each blood type of the donors. The group AB donors exhibited significantly higher FVIII and VWF:RCo in FFP and concentrated FFP compared to the group O donors, whereas the values of these coagulation factors in cryoprecipitate were not significantly correlated with ABO blood groups. Each bag of FFP, concentrated FFP, and cryoprecipitate showed similar level of fibrinogen and ROTEM-MCF regardless of blood type.

**Table 3:** Subset analysis of the levels of Factor VIII, VWF:Ag and VWF:RCo in FFP, ultrafiltration units, and cryoprecipitate from each blood type of donors.

	A type	B type	O type	AB type
FFP				
VWF:RCo (IU/m)	76.8 ± 15.9	104.4 ± 20.4	76.6 ± 25.4	116.4 ± 20.8*
VWF:Ag (IU/unit)	96.8 ± 24.3	131.4 ± 45.5	82.4 ± 18.5	144.6 ± 24.6*
FVIII (%)	96.6 ± 21.9	123.9 ± 26.5	82.8 ± 22.0	131.6 ± 37.7*
Fibrinogen (mg/dL)	217.6 ± 51.3	283.4 ± 99.8	309.0 ± 53.5	281.2 ± 43.8
EXTEM-MCF	18.5 ± 3.4	21.2 ± 7.1	24.6 ± 3.6	19.6 ± 2.5
NATEM-MCF	19.3 ± 2.2	23.0 ± 9.2	23.6 ± 3.8	22.0 ± 4.1
Ultrafiltration				
VWF:RCo (IU/m)	206.0 ± 72.8	234.6 ± 83.4	180.8 ± 24.0	296.8 ± 48.4*
VWF:Ag (IU/unit)	222.4 ± 77.0	310.2 ± 112,0	221.6 ± 44.1	348.4 ± 55.7
FVIII (%)	278.4 ± 57.3	314.2 ± 70.5	251.8 ± 62.5	390.5 ± 62.9*
Fibrinogen (mg/dL)	634.0 ± 94.7	788.4 ± 131.5	799.0 ± 98.5	646.0 ± 177.8
EXTEM-MCF	29.8 ± 2.9	38.6 ± 7.6	41.8 ± 9.2	34.4 ± 7.7
NATEM-MCF	30.5 ± 3.1	36.0 ± 5.8	37.2 ± 5.4	33.0 ± 7.0
Cryoprecipitate				
VWF:RCo (IU/m)	619.4 ± 145.2	641.8 ± 81.1	458.4 ± 160.7	741.0 ± 208.1
VWF:Ag (IU/unit)	874.8 ± 161.6	937.6 ± 289.8	609.0 ± 154.4	1040.8 ± 350.3
FVIII (%)	492.4 ± 157.8	593.7 ± 259.4	356.8 ± 142.9	504.5 ± 100.0
Fibrinogen (mg/dL)	> 900	> 900	> 900	> 900
EXTEM-MCF	65.4 ± 4.7	65.6 ± 14.7	66.6 ± 7.7	62.4 ± 13.0
NATEM-MCF	67.6 ± 7.3	65.8 ± 13.8	67.8 ± 9.7	65.6 ± 12.9

Values are expressed as mean ± SD. Differences compared to the group O donors (*) are shown if results from two-way analysis of variance for repeated measures are significant (P < 0.05).

*FFP* fresh frozen plasma, *VWF:RCo* von Willebrand factor ristocetin cofactor activity, *VWF:Ag* von Willebrand factor antigen, *PT-INR* prothrombin time-international normalized ratio, *PT* prothrombin time, *APTT* activated partial thromboplastin time, *EXTEM* extrinsic test, *CT* clotting time, *CFT* clot formation time, *MCF* maximum clot firmness, *NATEM* non-activated thromboelastometry, *NA* not assessed.

## Discussion

We found that after ultrafiltration, we increased fibrinogen concentration 2.7 fold higher, a level consistent with prior reports^12^ in patients where MUF was used for bleeding management following cardiac surgery^6–8^. Although concentrated FFP contains less fibrinogen than cryoprecipitate, it also provides multiple other important hemostatic factors that may be important for bleeding management^13,14^. After UF, 36.5% of fibrinogen (mean levels of 448 mg) was extracted from 480 mL of FFP, a level less than in cryoprecipitate, which usually contains 40–60% of fibrinogen levels from FFP^15^. We also noted a correlation between the fibrinogen content of the FFP and concentrated FFP after UF (*r* = 0.634, *p* < 0.0001), as a higher fibrinogen content of FFP influences the final levels. FFP with fibrinogen levels higher than 330 mg/dL was concentrated to levels over 900 mg/dL.

In addition to fibrinogen, increased levels of FV, FIX, and FXIII in the concentrated FFP may also improve hemostasis by activating tenase and prothrombinase to increase thrombin generation^16,17^ and polymerizing fibrin cross-linked α2 antiplasmin, thus making fibrin more resistant to degradation^18,19^. Another potential advantage of concentrated FFP is that it provides antithrombin and other plasma proteins, which may be significant for restoring hemostatic balance^20,21^.

Further, the increase in total proteins and albumin in concentrated FFP contributes to increasing colloid osmotic pressure^22–24^. Overall improvements in hemostasis with concentrated FFP occurred as noted by the shortened CFT, and the elevated α-angle of NATEM, and fibrin polymerization. However, EXTEM-CT was significantly prolonged after UF, which might be affected by concentrated anticoagulant (Acid Citrate Dextrose-A) in FFP. In contrast, NATEM-CT was significantly shortened with decreased PT, possibly due to TF generation during UF^25^. This discrepancy may be attributed to the sensitivity of viscoelastic tests to the initial activation of the coagulation cascade^26^. Because thrombin generation was amplified by increasing TF concentrations, the EXTEM test is reproducible, and results were comparable among patients^27,28^. In this study, the variability of NATEM-CT also decreased by adding TF in the EXTEM assay. However, NATEM analysis pronounced the changes in CFT and α-angle during UF, which were in agreement with other coagulation assay. This suggests that NATEM analysis could provide a more subtle depiction of the hemostatic status.

Despite the ABO blood group, labile FVIII and VWF, known as acute phase reactants^29^ were significantly higher in cryoprecipitate than in concentrated FFP. In addition, group O donors had significantly lower FVIII and VWF levels in FFP than group AB donors, findings consistent with other studies^30,31^. Theoretically, the ABO antigens may alter the rate of VWF synthesis, secretion^31^, or clearance^31^ accompanied by the changes in FVIII levels. The levels of FVIII and VWF:RCo were significantly different among ABO blood groups after UF, but were the same in the cryoprecipitate. However, blood types were not related to fibrinogen level and fibrin polymerization.

The difference in hemostatic potency and plasma properties between concentrated FFP and cryoprecipitate can be explained by separating fibrinogen and coagulation factors from FFP. Fibrinogen and other coagulation factors in cryoprecipitate were partially lost by the remaining plasma, called cryosupernatant^32^, and by activation or denaturation due to freezing temperature, increased pH^33^, and the technique itself^34^. However, the UF technique removes water, electrolyte, and small molecules across a semipermeable membrane by hydrostatic pressure^35^. Theoretically, the effect of filtration or adsorption^35^ on the ultrafilter membrane depends on the molecular weights of coagulation factors. The type of hemofilter used in this study has a cut-off point of 10 kDa, and substances with a molecular weight of less than 50 kDa can be removed by UF. However, larger molecular components (> 300 kDa), such as VWF, FVIII, FXIII, and fibrinogen^36^, were also not completely preserved. The direct flow in UF moves perpendicular to the membrane, resulting in fairing and concentration polarization^37,38^, which reduce the driving force within the membrane, deteriorate the selectivity of separation, and extends filtration time, although average lead time for processing was 32 min.

This study has several limitations. First, it was based on in vitro experiments conducted under static conditions. Second, more than 20 mL of aliquots were required after each removal of predefined plasma water. Especially pre-washing is necessary before manual measurement of the plasma density. Therefore, more fibrinogen would be extracted from FFP after UF. Finally, the extraction rates of fibrinogen and coagulation factors were specific to UF methods with a polysulfone-based membrane run by an ultrafilter pump. Many polysulfone-based membranes are combined with polyvinylpyrrolidone (PVP) to avoid protein adsorption, as described by the Vroman effect^39^. The state of PVP on the inner surface of membranes has an effect on preventing protein adsorption onto membrane surfaces^36,40^. Consequently, our findings may not be generalizable to other types of dialyzers.

In conclusion, UF could reduce the total volume and increase fibrinogen concentration 2.7 times within 32 min but did not reach similar levels of hemostatic factors in cryoprecipitate. Concentrated FFP could restore FV, FIX, and FXIII as well as antithrombin and provide additional plasma proteins. Blood types were not related to hemostatic potency, despite the different level of FVIII and VWF depending on ABO blood groups. Additional clinical effects of concentrated FFP may further determine its potential application for managing bleeding and cardiac surgical patients when cryoprecipitate is not readily available. Finally, this in vitro study offers insights into UF, but its potential effects need to be further investigated in clinical studies.

## Methods

### Outline of the study

We used ABO-specific FFP-LR480 (480 mL) supplied by the Japan Red Cross because plasma levels of VWF and FVIII are different among ABO blood group. Five units of FFP from each blood type were used for making UF concentrate, and equal FFP units were used to produce cryoprecipitate. The final volume of concentrated FFP after 250 mL removal was comparable to that of cryoprecipitate. The validation study was performed in samples after removing 50, 100, 200, and 250 mL of fluid volume from FFP by (1) evaluating the change of prothrombin time (PT), activated partial thromboplastin time (APTT), fibrinogen, antithrombin activity, coagulation factors, VWF:antigen (Ag), activity, and viscoelastic properties as well as, total protein, and albumin, density and osmotic pressure; (2) comparing of the coagulation parameters, plasma proteins, and plasma properties, such as density and osmotic pressure between cryoprecipitate at the time of thaw and the concentrated FFP after 250 mL fluid removal. Our Institutional Review Board (No. 5433) approved this study, and informed consent was obtained as an opt-out function on the website.

### Preparation of concentrated FFP by ultrafiltration

**Figure 1:**
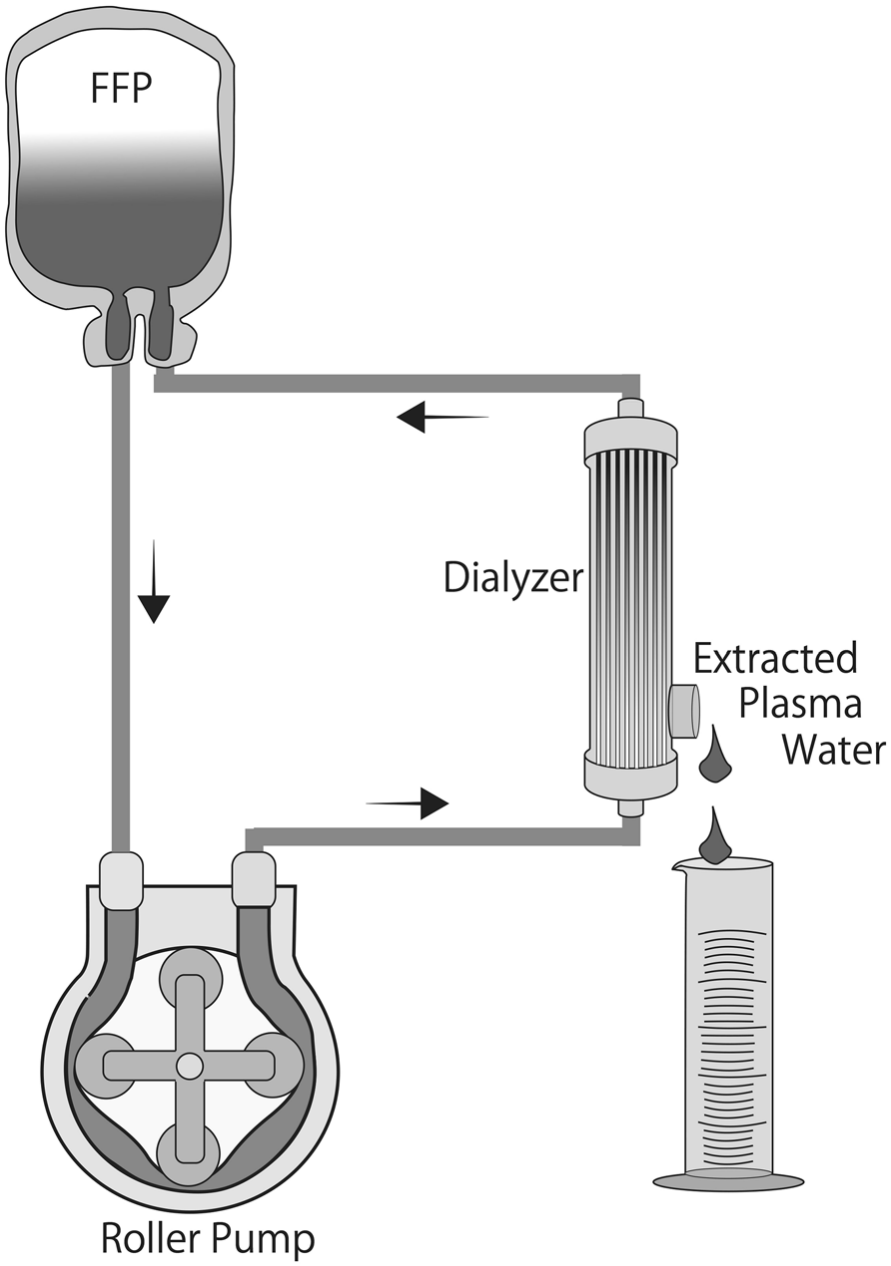
Schematic representation of the ultrafiltration circuit for concentrating fibrinogen and other coagulation factors in FFP using an ultrafilter pump. FFP was circulated using a dialyzer with a polysulfone-based membrane to remove plasma fluid through a convective process involving filtration across membranes. The ultrafilter pump ran at a flow rate of 300 mL/min to remove plasma water using a hollow fiber.

FFPs were thawed at 37 °C and circulated through a dialyzer with a polysulfone-based membrane (NV-10U: Toray, Tokyo, Japan, an inner diameter of 200 μm, a wall thickness of 40 μm, Gamma-ray sterilization, and Extrapolation rate of 43 mL/h/mmHg), which was a similar type of hemofilter used during CPB, to remove fluid through a convective process involving filtration across membranes (Fig. 1). The ultrafilter pump ran at a flow rate of 300 mL/min for ultrafiltrate removal with a hollow fiber, which is equivalent to UF’s flow rate in adults during CPB. The amount of plasma water extracted was measured, and the aliquot from each concentrated FFP was used for the measurements.

### Preparation of cryoprecipitate

Cryoprecipitate was prepared from a single-donor FFP that was thawed at 1–6 °C for 12–16 h and then subjected to a hard spin at 4500*g* for a 10 min centrifugation^34,41^. The supernatant plasma was removed, leaving an insoluble precipitate and a reduced plasma volume. The residual material was resuspended, refrozen within 1 h of thawing, and stored in the blast freezer at − 18 °C. Cryoprecipitate samples were collected for laboratory and viscoelastic measurements after thawing in batches at 30–37 °C.

### Laboratory measurements

While total protein and albumin were measured using Labospect008α (Hitachi, Tokyo, Japan), coagulation profiles, including PT, aPTT, PT-international normalized ratio, fibrinogen level (Clauss method), and antithrombin activity, were analyzed in the central hematological laboratory using XN-3000 (Sysmex Co., Kobe, Japan), according to the institutional protocol. The activity of FV, FVIII, and IX and the activity of FXIII were determined with CS-5100 (Sysmex, Kobe, Japan) using a one-stage clotting assay for individual factor-deficient plasma (Siemens Healthineers, Marburg, Germany) and JCA-BM8020 (JEOL, Tokyo, Japan) using synthetic substrate method with Berichrom F XIII (Sysmex, Kobe, Japan), respectively. While VWF:Ag was tested by ACL-TOP 700 (Werfen, Barcelona, Spain) using latex immunoturbidometric assay (HrmosIL von Willebrand Factor Antigen; Instrumentation Laboratory Company), VWF activity was assessed as VWF:ristocetin cofactor activity (VWF:RCo) with CS-5100 (Sysmex, Kobe, Japan) using aggregometry with lyophilized fixed platelets and ristocetin (BC von Willebrand reagent; Simens Healthcare Diagnostics). The plasma density was measured by Density/Specific Gravity Meter (DA-130N: Kyoto Electronics manufacturing, Kyoto, Japan), which detects oscillation specific to a substance in proportion to the weight mass. Osmotic pressure was determined using freezing point descent methods with OsmoStaison (Arkray, Kyoto, Japan).

### The viscoelastic properties of plasma

Clot formation was induced by adding 20 μL of 0.2 M calcium chloride solution (starTEM 20 reagent, TemInnoations GmbH, Munich, Germany). The reagent was added to the cup and mixed adequately with 300 μL of plasma sample. We performed thromboelastometry without adding additional activators (NATEM), as well as adding recombinant tissue factor-(TF), and phospholipid-activated ROTEM (EXTEM). The following ROTEM parameters were analyzed: the clotting time (CT [s]), alpha angle (angle of the tangent at a 2-mm amplitude [°]), the clot formation time (CFT [s]), and the maximum clot firmness (MCF [mm]).

### Statistical analyses

Data were tested for normal distribution using the Shapiro‒Wilk test. Either repeated measures one-way analysis of variance, or Friedman test was performed to detect changes in coagulation profiles, plasma proteins and ROTEM values between FFP and after each hemoconcentration. The various parameters obtained from cryoprecipitate and concentrated FFP were compared using the two-tailed Student’s *t*-test or Mann–Whitney’s *U* test, depending on the underlying distribution. The differences in FVIII, VWF:Ag, and VWF:RCo in FFP, cryoprecipitate, and concentrated FFP from each blood type were assessed by analysis of variance with post hoc Tukey test. The criterion for rejection of the null hypothesis was p < 0.05. All statistical analyses, except statistical power analyses, were performed using the Statistical Package for the Social Sciences software (version 11.0; IBM, Chicago, IL, USA).

### Sample size calculation

We conducted a post hoc power analysis using G*Power 3.1 based on the result of a study that showed a significant difference in MCF values between 20 units of cryoprecipitate and 20 units of concentrated FFP using an unpaired t-test.

### Supplementary Information

Supplementary Information.

## Data Availability

All data generated or analysed during this study are included in this published article and its Supplementary Information Files.
